# A BAC-bacterial recombination method to generate physically linked multiple gene reporter DNA constructs

**DOI:** 10.1186/1472-6750-9-20

**Published:** 2009-03-13

**Authors:** Peter Maye, Mary Louise Stover, Yaling Liu, David W Rowe, Shiaoching Gong, Alexander C Lichtler

**Affiliations:** 1 Department of Reconstructive Sciences, Center for Regenerative Medicine, University of Connecticut Health Center, Farmington, CT, USA; 2 GENSAT Project, Rockefeller University, New York, New York, USA

## Abstract

**Background:**

Reporter gene mice are valuable animal models for biological research providing a gene expression readout that can contribute to cellular characterization within the context of a developmental process. With the advancement of bacterial recombination techniques to engineer reporter gene constructs from BAC genomic clones and the generation of optically distinguishable fluorescent protein reporter genes, there is an unprecedented capability to engineer more informative transgenic reporter mouse models relative to what has been traditionally available.

**Results:**

We demonstrate here our first effort on the development of a three stage bacterial recombination strategy to physically link multiple genes together with their respective fluorescent protein (FP) reporters in one DNA fragment. This strategy uses bacterial recombination techniques to: (1) subclone genes of interest into BAC linking vectors, (2) insert desired reporter genes into respective genes and (3) link different gene-reporters together. As proof of concept, we have generated a single DNA fragment containing the genes *Trap*, *Dmp1*, and *Ibsp *driving the expression of ECFP, mCherry, and Topaz FP reporter genes, respectively. Using this DNA construct, we have successfully generated transgenic reporter mice that retain two to three gene readouts.

**Conclusion:**

The three stage methodology to link multiple genes with their respective fluorescent protein reporter works with reasonable efficiency. Moreover, gene linkage allows for their common chromosomal integration into a single locus. However, the testing of this multi-reporter DNA construct by transgenesis does suggest that the linkage of two different genes together, despite their large size, can still create a positional effect. We believe that gene choice, genomic DNA fragment size and the presence of endogenous insulator elements are critical variables.

## Background

To better define cell types of the bone lineage, our past work has exploited the use of fluorescent protein (FP) reporter gene mice [1-4]. This work has involved standard transgenic approaches where defined transcriptional regulatory regions derived from genes selectively expressed in bone cells drive the expression of a reporter gene to mark distinct cell types. By elegant mutagenesis schemes and directed evolution, the generation of FP variants has expanded considerably and covers the visible spectrum [5]. Among these different FP variants there are at least three colors that are optically separable, cyan, yellow, and red. This allows for multiplexing, where multiple FP readouts can be combined and viewed simultaneously, but distinctly detected. Multiplexing approaches are advantageous for a variety of reasons including, (1) the ability to undertake combinatorial biological approaches where different reporter readouts allow the association of different biological events, (2) multiple FP readouts can further resolve molecular mechanisms, and (3) data can be quickly acquired from multiple readouts in the same sample.

To capitalize on the separable nature of FP reporters we have generated transgenic mouse lines containing different FP spectral variants allowing us to cross two or more mouse lines together to further define bone cell populations [6]. We envision an even greater role for FP reporter mice to aid in the investigation of complex biological mechanisms. Unfortunately, we are limited in the pace of research and the types of questions we can answer as a result of the one promoter-reporter gene/mouse model. While breeding two distinct transgenic mouse lines together to visualize two distinct reporter genes in the same mouse is straightforward, adding a third variable, such as a genetic mutation, becomes dramatically more time consuming, and adding a fourth variable, such as a cre recombinase, often makes the experiment unrealistic to carry out. Therefore, while multiplexing strategies are highly desirable, it can also be impractical to use reporter gene mice in a research study when the breeding schemes of combining different genetic loci into the same animal become too time consuming and costly.

This problem has made us reconsider the design of transgenic reporter gene mice and appreciate the great value in establishing a methodology that would result in the generation of a single DNA fragment containing multiple reporter gene elements. This DNA construct could then be used for mouse transgenesis to create an animal model where the multiple reporter genes would insert into a single locus, thus simplifying breeding schemes, yet expanding the capability and usage of the animal model. Ideally, the expression of the different reporter genes should not influence each other and accurately represent their respective endogenous gene's expression.

Within the past decade there have been notable advances in the genetic engineering of mice including the use of homologous recombination in bacteria to engineer BAC cloned genomic DNA fragments for mouse transgenesis [7-9]. BACs hold large fragments of genomic DNA (~200 KB) sometimes containing more than one gene. An indirect benefit of genome sequencing projects has been the careful annotation of BAC genomic clones allowing investigators to obtain specific clones without laborious screening. For most genes of interest, BACs can be chosen that are likely to contain all the transcriptional regulatory elements. As a result, reporter gene expression in transgenic mice, generated from large BAC genomic inserts, accurately reflects endogenous gene expression. Importantly, a variety of bacterial recombination systems exist to modify genomic DNA in bacteria, including those that employ *RecA *or the Red genes from bacteriophage lambda [7-9]. Strategies to modify genomic DNA fragments include the ability to insert reporter genes into BAC cloned genes and the physical linkage of two overlapping BAC clones [10].

Based on these existing methodologies, we have pursued a combinatorial strategy composed of inserting spectrally distinct FP reporter genes into genes of interest followed by linking these gene fragments together. As proof of principle and in an effort to generate an animal model to mark multiple cell types relevant to bone biology, we have subcloned two genomic DNA fragments into BAC linking vectors GM and SP. One genomic DNA fragment contains the gene *Acid Phosphatase 5 *(*Acp5*), better known as *Tartrate Resistant Acid Phosphatase *(*Trap*), which is expressed in osteoclasts [11,12]. The second genomic DNA fragment contains the genes *Dentin Matrix Protein-1 *(*Dmp-1*) and *Integrin Binding Sialoprotein *(*Ibsp*), which are highly expressed in osteocytes and osteoblasts, respectively [13-18]. FP reporters ECFP, mCherry, and the YFP variant, Topaz were inserted into *Trap*, *Dmp-1 *and *Ibsp*, respectively. These two gene fragments were then linked together and transgenic founders were generated. We report here on our methodology and present some preliminary characterization of different founder lines towards advancing this technology.

## Results

### Subcloning Genes into BAC Linking Vectors

**Figure 1:**
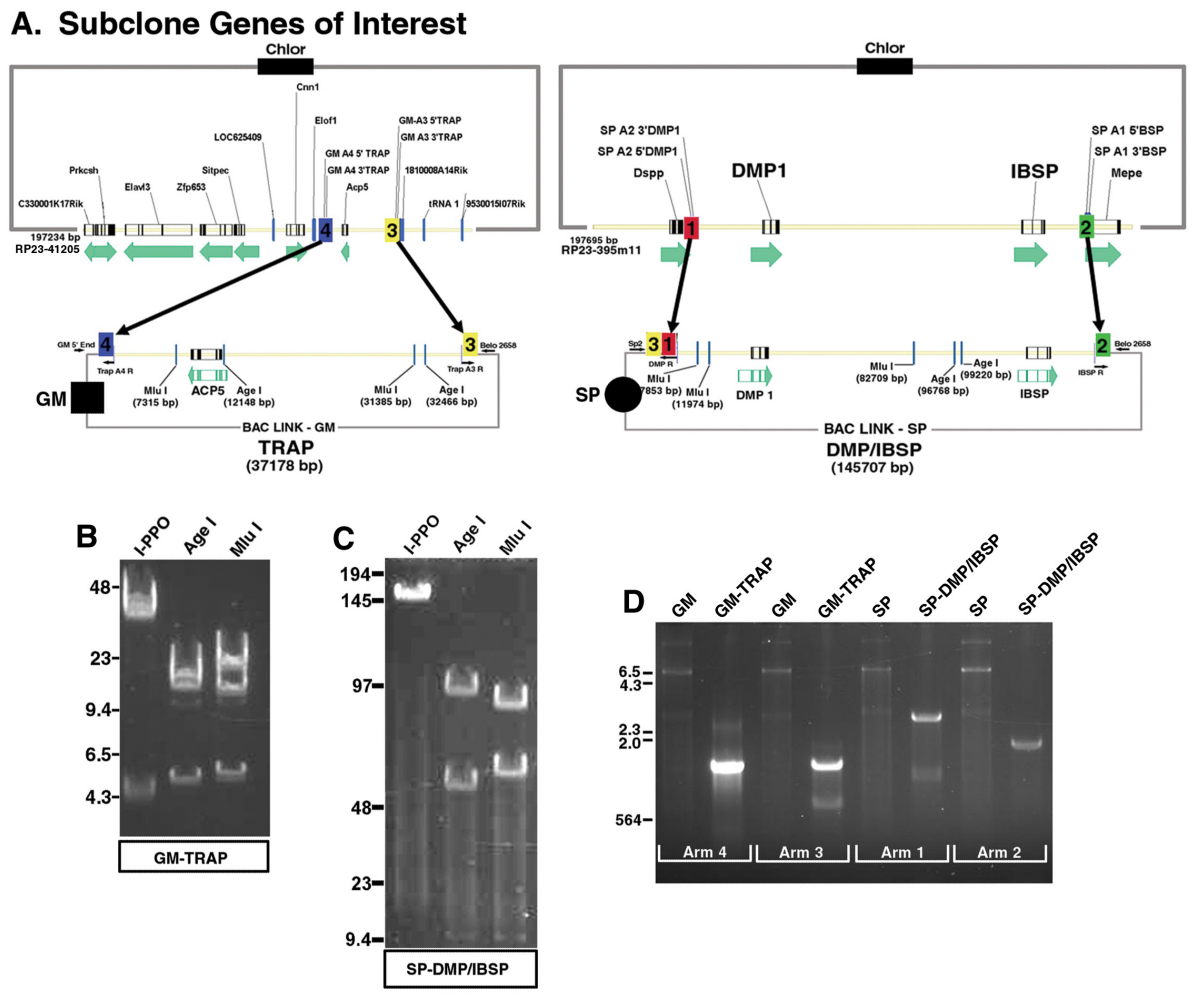
**Step 1: Subclone genes of interest**. (A) Diagram showing the subcloning of defined genomic DNA regions from BAC clones into BACLinking vectors. RP23-395m11 containing the genes *Dmp1 *and *Ibsp *was subcloned into BACLink SP and RP23-41205 containing the *Trap *gene was subcloned into BACLink GM. Homology arm pairs are indicated by 1 & 2 for *Dmp1/Ibsp *and 3 & 4 for *Trap*. (B-D) Confirmation of subcloned genomic DNA fragments. (B&C) Diagnostic restriction digests of subcloned GM-*Trap *and SP-*Dmp1/Ibsp *were carried out using I-PPO1, Age1, and Mlu1. (B) For GM-*Trap*, I-PPO1 releases the whole insert ~37 KB + partial vector sequences. Two I-PPO1 sites flank the belo origin of replication which shows up as a ~4.5 KB DNA fragment. Restriction digestion with Age1 yielded DNA fragment sizes 20.3 KB, 12.1 KB+partial vector sequences, and 4.7+partial vector sequences. Restriction digestion with Mlu1 yielded DNA fragment sizes ~24 KB, 7.3 KB+ vector backbone, and 5.8 KB. DNA sequence is not available for BACLink-GM, however, we estimate the vector backbone to be ~6.5 KB. (C) For SP-*Dmp1/Ibsp *I-PPO1 released the whole insert ~145 KB (Belo replication origin not shown). Restriction digestion with Age1 yielded DNA fragment sizes 96.7 KB+partial vector sequences, 46.4+partial vector sequences and 2.4 KB (not shown). Restriction digestion with Mlu1 yielded DNA fragment sizes of 70.7 KB, 63 KB+partial vector sequences, 7.8 KB + partial vector sequences, and 4.1 KB (not shown). DNA sequence information for BACLink SP is not available, but we estimate the vector backbone to be similar to BACLink GM (~6.5 KB). (D) Diagnostic PCR was carried out with primers that flanked the homology arms. PCR amplification of BAC ends gave predicted sizes for homology arms 3 and 4, which was >1 KB. PCR amplification of Arm1 is larger than normal because this region also includes arm 3 (>2 KB in size). PCR amplification of arm 2 downstream of *Ibsp *did produce a product larger than expected, but does indicate that recombination did not occur too far downstream of our target site.

Past work has resulted in the generation of BAC linking vectors BACLink-GM, and BACLink-SP for the purpose of combining the components of one large gene, whose overlapping regions extended over multiple BAC clones, into one BAC vector [10]. This original linkage design relied on regions of homology, one of which was common to both genomic inserts and a second region present in the vector backbone. Importantly, these regions of homology flanked different antibiotic resistance sequences, gentamicin (GM) and spectinomycin (SP), allowing selection for the desired clone after homologous recombination. We reasoned that this strategy could also be used to link different genes of interest together, by engineering a second homology arm common to both BAC linking vectors (arm 3, Fig. 1A).

To subclone genes of interest, BAC clones RP23-412O5 and RP23-395M11 containing the genes for *Trap *and *Dmp1*/*Ibsp*, respectively were obtained from Children's Hospital Oakland Research Institute (CHORI) and their sequence identity verified. We decided to link two separate genomic DNA fragments containing a total of three genes. One DNA fragment contained the gene encoding for TRAP, an osteoclast marker, while the second DNA fragment contained the genes *Dmp1 *and *Ibsp*, whose protein products are highly expressed in osteocytes and osteoblasts, respectively. *Trap *is located on chromosome 9, while *Dmp1 *and *Ibsp *are adjacent to each other on mouse chromosome 5. To link the *Trap *genomic fragment with the *Dmp1*/*Ibsp *genomic fragment we cloned three homology arms, each ~1 kb in size into BACLink-SP (Arms 1, 2, & 3) and two homology arms were cloned into BACLink-GM (Arms 3 & 4). Arms 1 & 2 flanked a ~145 KB DNA region upstream of *Dmp1 *and downstream of *Ibsp*, while Arms 3 & 4 flanked a ~37 KB DNA region of *Trap *(Fig. 1A, B, &1C). *Trap *homology Arm3 was cloned into both BACLinking vectors as the second common homology arm in addition to a region of the chloramphenicol acetyltransferase coding sequence that is present in the vector backbone of both linking vectors. Based on past work indicating that the size limit for linkage was between 50–60 kb, the *Trap *genomic fragment was chosen for linkage. However, it is worth mentioning for larger genes, successive rounds of linkage can be carried out [10]. The *Dmp1*/*Ibsp *subcloned region started 26635 bp upstream of the *Dmp1 *translational start codon and went to 15,596 bp downstream of the *Ibsp *stop codon. While the subcloned region of *Trap *started 25,256 bp upstream of the *Trap *translation start codon and went to 8,813 bp downstream of the *Trap *stop codon.

To carry out subcloning, the bacteriophage λ Red recombinase system was introduced into host bacteria containing RP23-41205 and RP-23-395m11 by using the previously described mini λ vector [19]. BACLink vectors, were restriction digested within their homology arms, transformed, and selected for with gentamicin (*Trap*) or spectinomycin (*Dmp1*/*Ibsp*). Subcloned genomic fragments were confirmed by restriction digestion and field inversion gel electrophoresis (FIGE) (Fig. 1B &1C) and also by PCR amplification of BAC ends (Fig. 1D). GM-*Trap *and SP-*Dmp1*/*Ibsp *were digested with I-PPO1, Age I, and Mlu I. Restriction digestion of GM-*Trap *and GM-*Dmp1*/*Ibsp *with I-PPO1 yielded DNA fragments that corresponded well with the predicted insert size of ~37 KB for *Trap *and ~145 KB for *Dmp1*/*Ibsp *(Fig. 1B &1C). Further analysis of subcloned vectors with Age I and Mlu I also produced DNA fragments of predicted size (Fig. 1B &1C; please see figure legend for details). To confirm the accuracy of homologous recombination, PCR was carried out using primer sets that flanked homology arms to confirm the genomic insert ends (Fig. 1D). The subcloning procedure retained an efficiency of 20%.

### Inserting Fluorescent Protein Reporters

**Figure 2:**
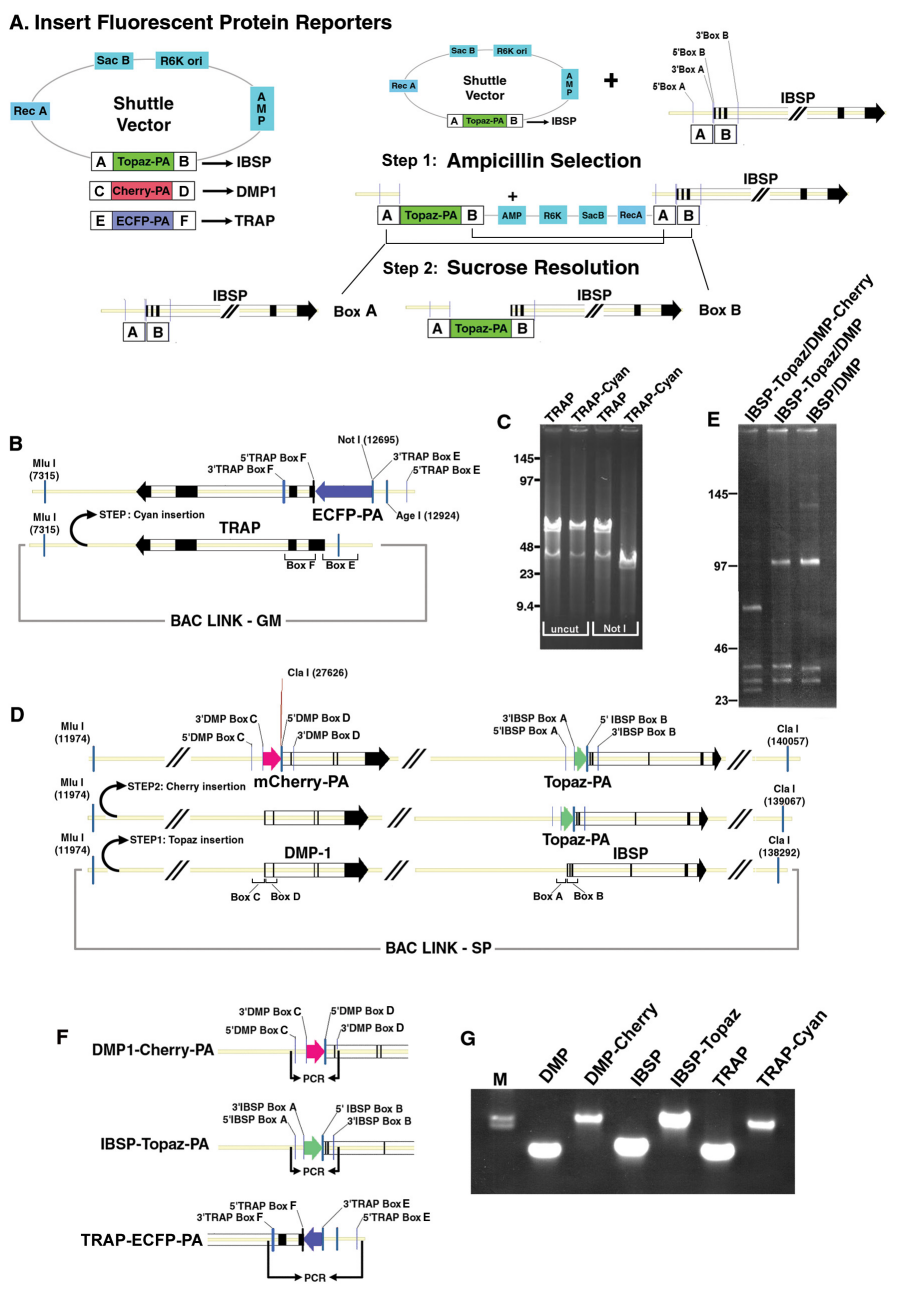
**Step 2: Insertion of fluorescent protein reporter genes**. (A) Diagrams showing the shuttle vectors containing different spectral FP variants for the specified genes and the two step recombination methodology. Homology arms for *Ibsp *(Arms A & B), *Dmp1 *(Arms C & D) and *Trap *(Arms E & F) were cloned into pLD53-Topaz, pLD53-mCherry, and pLD53-ECFP. Recombination was selected for using ampicillin plus gentamicin or spectinomycin. Sucrose resolution was carried out to remove unwanted vector sequences including ampicillin resistance. (B) Diagram of ECFP insertion into TRAP. (C) ECFP insertion introduced a unique Not1 site verifying integration into GM-*Trap*. (D) Diagram of Topaz insertion into *Ibsp *followed by mCherry insertion into *Dmp1*. (E) Cla1 restriction digestion of *Dmp1/Ibsp *subcloned fragment at progressive stages of reporter gene insertion. The insertion of mCherry introduces an additional Cla1 site. (F) Diagram of diagnostic PCR carried out with primers flanking the homology arms and fluorescent protein coding regions for all three genes. (G) PCR amplification comparing original BAC clones to fluorescent protein reporter BAC clones. PCR product sizes without reporter gene insertion were 1426 bp for *Dmp1*, 1569 bp for *Ibsp*, and 1450 bp for *Trap*. PCR product sizes with reporter gene insertion were 2559 bp for *Dmp1*, 2299 bp for *Ibsp*, and 2545 bp for *Trap*.

**Figure 3:**
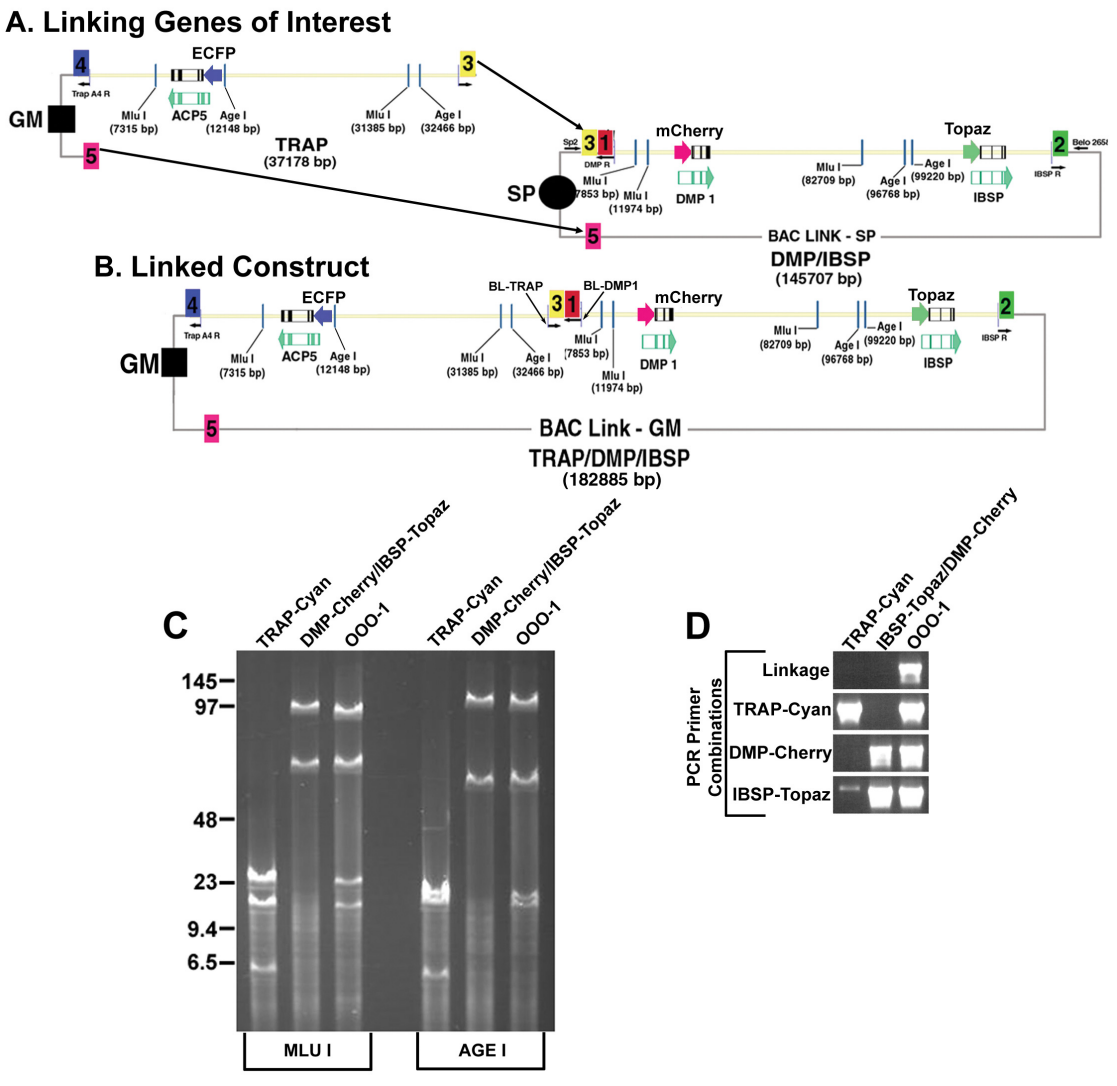
**Step 3: Linking genomic regions**. (A & B) Diagram showing the linkage of BACLink *Trap *GM into BACLink *Dmp1/Ibsp *SP. BACLink *Trap *GM was digested with I-PPO1 exposing homology arms 3 (*Trap *homology arm) and 5 (endogenous homology arm to both GM and SP vectors) and electroporated into bacteria containing BACLink *Dmp1/Ibsp *SP containing the Red recombinase system. (B) The newly linked vector changes from SP resistant to GM resistant. BL-TRAP and BL-DMP1 demarcate primer sites that flank the linked region and were used as a primary screen. (C & D) Verification of linked BAC construct (OOO-1) compared to the individually generated GM-*Trap*-ECFP and SP-*Dmp1*-mCherry/*Ibsp*-Topaz. (C) Mlu1 and Age1 diagnostic restriction digest shows a banding pattern consistent with its original BAC clone counterparts. (D) Diagnostic PCR amplification of the linked genomic region and individual fluorescent protein reporters confirming the existence of all three fluorescent reporter genes in the linked BAC DNA construct.

Different bacterial recombination strategies exist to insert a reporter gene into a gene of interest. We favored using a two step recombination strategy developed by Heintz and co-workers (Fig. 2A) because it involves a resolution step to remove unwanted vector sequences and antibiotic selection genes allowing us to repeatedly target multiple genes within the same genomic DNA fragment [8]. The shuttle vector (diagramed in Fig. 2A) has positive (ampicillin) and negative (Sac B) selection schemes that allows for insertion of the vector and resolution of unwanted vector sequences in two consecutive recombination steps (Fig. 2A). We modified the original pLD53-SCAEB vector by changing the original EGFP with the spectrally distinct FP reporters, Topaz, mCherry, and ECFP (Fig. 2A). To avoid the creation of undesireable regions of homology, we also decided to engineer our FP reporters with three different polyadenylation sequences, SV40, bovine growth hormone, and β-globin for ECFP, Topaz, and mCherry, respectively. Furthermore, we decided to keep Topaz and ECFP, *Aequorea victoria *GFP variants, which have nearly identical DNA sequences, separate by recombining them into the two different BAC subclones. Recombination between Topaz and ECFP during linkage (Fig. 3) is less of concern since this step involves the use of the Red recombinase system, which works by a gap end repair mechanism.

To insert FP reporters, two distinct homology arms (*Ibsp *arms A & B), (*Dmp1 *arms C & D), and (*Trap *arms E & F) were PCR amplified for each gene from the original BAC clones and cloned into Topaz, mCherry, and ECFP shuttle vectors. To avoid problems with the reporter gene reading frame, the homology arm preceding the FP reporter (in this case, A and C) was located a few nucleotides before the gene's endogenous translational start site. However, for *Trap *the translational start site overlaps with a splice acceptor site, therefore homology arm E extends +12 bp past the translational start site in the same reading frame as ECFP. The shuttle vector two step recombination strategy was carried out for all three genes and is diagrammatically shown for *Ibsp *(Fig. 2A). Shuttle vectors were transformed into host bacteria containing either GM-*Trap *or SP-*Dmp1*/*Ibsp *subclones and underwent amp/GM or amp/SP selection. Ampicillin resistant colonies were screened by colony PCR for the insertion event. Positive colonies were plated on sucrose plates to resolve vector sequences and screened again by PCR. PCR screens for resolution used primer sets that flanked outer homology arms (Fig. 2F). FP reporter insertion resulted in a notably larger PCR product (Fig. 2G, amplicon sizes are provided in the figure legend). Positive clones were further analyzed by restriction digestion. Insertion of ECFP-PA into the *Trap *gene introduced a unique NotI site (Fig. 2C). Since the SP subclone contains two genes of interest, two consecutive rounds of recombination had to be carried out first using the Topaz shuttle vector, then the mCherry shuttle vector. Insertion of mCherry-PA into *Dmp1 *introduced an additional Cla I site (Fig. 2E).

### Linking Genes of Interest

Prior to subcloning the *Dmp1*/*Ibsp *genomic fragment into BACLink-SP, a homology arm for *Trap *(Arm3) was cloned into BACLink-SP to allow future linkage of the *Trap *genomic DNA fragment to the *Dmp1*/*Ibsp *genomic fragment. For linkage we added the Red recombinase system to the host bacteria containing the SP-Dmp *1*-mCherry/Ibsp-Topaz genomic DNA fragment by transformation with mini-λ DNA [19]. GM-Trap-ECFP was then digested with I-PPO and transformed into the host bacteria containing *Dmp1/Ibsp*. Linkage was selected for with gentamicin, since during linkage spectinomycin resistance will be lost and exchanged for gentamicin resistance (DNA constructs diagramed in Figure 3A &3B, not to scale). Linked products were initially screened by PCR using primers against *Trap *and *Dmp1 *(BL-*TRAP*/BL-*DMP1*). From the gentamicin resistant colonies, we determined that this linkage step had an efficiency of 10%. Positive colonies were further analyzed by restriction enzyme analysis with Mlu I and Age I to confirm linkage and the size of digested products. Figure 3C is a diagnostic restriction digest of the individual BACLink subclones, Trap-GM and Dmp-1/Ibsp-SP compared to the linked DNA construct (OOO-1), where the restriction digest confirms that the linked construct retains all the appropriate size DNA fragments that collectively are present in the two separate genomic subclones. We further confirmed linkage by comparing the PCR amplification of the three fluorescent protein reporters and the linkage region in separate subclones to the linked DNA construct Figure 3D.

### Functionality of Linked Reporter Construct in Transgenic Mice

**Figure 4:**
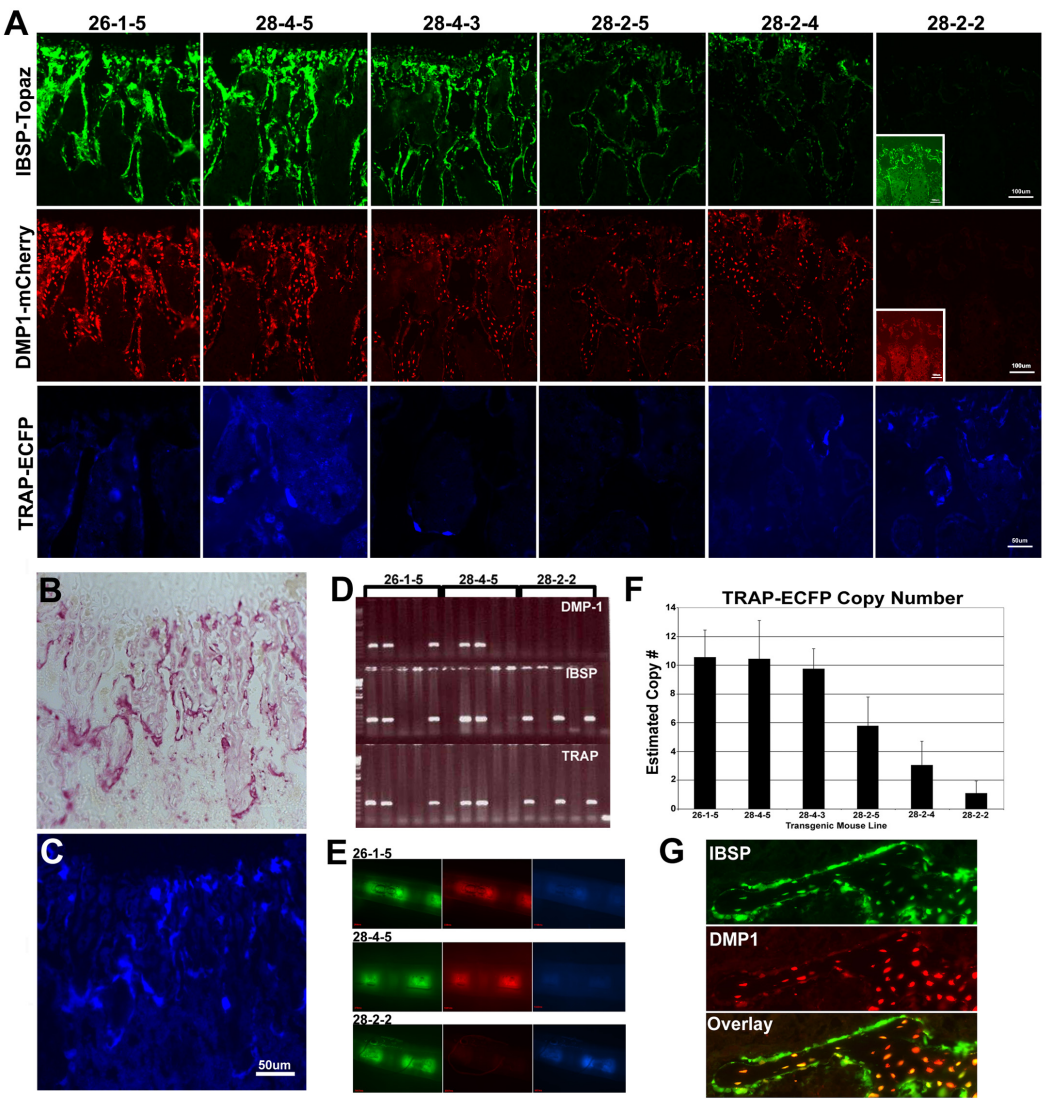
**Functional characterization of linked multi-reporter gene construct in transgenic mice**. (A) Imaging of fluorescent reporters in six week old spine under fixed exposure demonstrating intensity differences among different lines. *Ibsp*-Topaz and *Dmp1*-mCherry fluorescent reporter genes are easily detected in most lines, however *Trap*-ECFP in most lines appears highly mosaic expression with only a random cell having ECFP fluorescence. Line 28-2-2 is the only line that has uniform *Trap*-ECFP reporter expression, but it has no reporter expression for *Dmp1 *and low expression for *Ibsp*-Topaz (over exposed inset). (B&C) Confirming the fidelity of the *Trap*-ECFP reporter. (B) TRAP histological stain on a 3 week old femur that was initially imaged (C) for *Trap*-ECFP expression. ECFP expression and TRAP staining are essentially identical. (D-E) Genotyping of litters from three separate mouse lines for all three reporter genes. Individual reporters co-segregate together verifying common chromosomal integration. For lines 26-1-5 and 28-4-5 all three reporters are detected, however, in line 28-2-2 DMP1-mCherry reporter is not detected by PCR (D) or viewing of tail biopsies (E). (F) Estimated copy number of DNA construct in different transgenic lines using primers directed against the TRAP reporter. (G) High magnification expression in *Ibsp*-Topaz and *Dmp1*-mCherry in trabecular bone of a 6 week old female femur from line 28-4-3.

To determine the functionality of the linked three gene reporter construct, transgenic mice were generated by DNA pronuclear injection. Nine of thirty potential founder mice showed expression of one or more of the three reporters by examining the tail biopsy under a standard epifluorescence microscope. Three transgenic founders expressed all reporter genes at a very low level and therefore were euthanized. F2 generation animals from the six remaining transgenic founder lines are currently being characterized and future work will report on their properties in greater detail. However, our preliminary observations of these transgenic lines suggest the outcome of this technical approach to design multiple reporter gene constructs has met with mixed success. Imaging of 6 week old female spines across multiple lines under the same exposure conditions within each reporter demonstrates that F2 animals from lines 26-1-5, 28-4-3, 28-4-5, 28-2-4, 28-2-5 express *Ibsp*-Topaz and *Dmp1*-mCherry relatively strong while showing lower to undetectable levels of *Trap*-ECFP expression (Fig. 4A). Occasionally, a *Trap*-ECFP positive cell can be observed suggesting mosaic expression of the *Trap *reporter (Fig. 4A, *Trap*-ECFP). In contrast, line 28-2-2 expresses *Trap*-ECFP at higher levels and uniformly in osteoclasts as determined by comparing histological TRAP staining to *Trap*-ECFP (compare Fig. 4B to 4C), while showing low expression of *Ibsp *and no detectable expression of *Dmp1 *(Fig. 4A, inset images).

The detection of higher *Trap *reporter gene expression in line 28-2-2 was an interesting observation. We anticipated that the *Trap *gene, encoding for an acid phosphatase, would be expressed at lower levels relative to *Ibsp *and *Dmp1*, highly expressed sialoproteins. In agreement with this thinking, most of the transgenic lines generated from this construct resulted in much high reporter expression from *Ibsp *and *Dmp1*, than *Trap*. At the same time, we considered that the transcriptional regulation of *Trap*, taking place in an osteoclast, would be drastically different from *Dmp1 *and *Ibsp*, which are expressed in bone cells. Therefore, when 28-2-2 showed higher *Trap *reporter expression levels relative to all the other lines, we speculated that the transgenic construct integrated into a more favorable chromosomal site for *Trap *expression relative to *Dmp1 *and *Ibsp*. However, genotyping for each reporter gene within the transgenic construct surprisingly showed that while lines 26-1-5, 28-4-3, 28-4-5, 28-2-4, and 28-2-5 carried all three reporter genes that segregated together into the same offspring (Fig. 4D), we were unable to amplify *Dmp1*-mCherry in line 28-2-2 (Fig. 4D). Consistent with this genotyping data, we never observed *Dmp1*-mCherry expression in line 28-2-2, but did observe weak *Ibsp*-Topaz expression (Fig. 4A &4E, tail images). Since *Dmp-1 *mCherry is present in the middle of the transgenic construct, the likely explanation is that the transgenic construct that generated line 28-2-2 fragmented prior to chromosomal integration. If we also consider that separation of the *Trap *reporter from the *Dmp1 *and *Ibsp *genomic region also could have contributed to its increased *Trap *reporter expression, this then suggests that by linking *Trap *to *Dmp1 *and *Ibsp*, we may have created a positional effect that ultimately dampened *Trap *activity. However, it is also possible that the transgene copy number of *Trap*-ECFP is greater in line 28-2-2 relative to the other transgenic lines. To discern this possibility we used real time PCR to estimate transgene copy number for the *Trap*-ECFP reporter. However, from this analysis line 28-2-2 actually had the lowest estimated copy number for the *Trap *transgene (Fig. 4F). Moreover, transgene copy number analysis corresponded well with reporter gene expression for *Dmp1*-mCherry and *Ibsp*-Topaz (Fig. 4A).

Despite the less than ideal functioning of the *Trap *reporter, our preliminary observations indicate that the *Dmp1 *and *Ibsp *reporter gene readouts appear to be consistent with previous expression studies for these two genes in skeletal elements [13-16,20]. *Dmp1*-mCherry is highly expressed in osteocytes, but can also be detected at low levels in certain populations of osteoblasts and hypertrophic chondrocytes (Fig. 4G and data not shown). *Ibsp*-Topaz is highly expressed in osteoblasts, but also can be detected at lower levels in osteocytes and hypertrophic chondrocytes (Fig. 4G and data not shown). Therefore, we believe this animal model still has great utility for skeletal research.

## Discussion

Reporter gene mice are valuable animal models that can dramatically facilitate and enhance biological studies. In this study, we have exploited the development of optically distinct fluorescent protein reporters and bacterial recombination strategies to engineer multiple BAC reporters as one contiguous DNA fragment for the purposes of creating more informative and biologically relevant animal models. We have also tested the functionality of this multi-reporter transgenic construct by transgenesis in mice. While the analysis of these transgenic lines is on going, our preliminary findings have been very informative regarding variables to consider when approaching this methodology.

The technical scheme employed here has utilized previously described methodologies that we have adopted and carried out in a defined order containing three successive steps, (1) subcloning genes of interest into BACLinking vectors, (2) inserting fluorescent protein reporter genes, and (3) linking genes together. The recombination steps for each step of this process worked with reasonable efficiency, where frequency of recombinants ranged from 10–20% of the clones screened. For efficient subcloning of large genomic DNA fragments into the BACLinking vectors, we generated homology arms of roughly 1 KB in size and carried out restriction digests within the homology arm, not at the end of the homology arm using an artificially introduced restriction site. Furthermore, the suggested convention when designing homology arm A is to override the endogenous genes translational start. However, for some genes, such as *Trap*, the translation start also serves as the splice acceptor site, therefore, our first efforts to engineer a *Trap *reporter failed because when we recombined in the ECFP reporter we also destroyed the splice acceptor site.

The use of fluorescent protein reporters has great value allowing the visualization of gene expression under live conditions. With the generation of different spectral variants it is possible to multiplex at least three different fluorescent protein reporters using a standard epifluorescent microscope. In this study we have used ECFP (blue), Topaz (yellow), and mCherry (red) fluorescent protein reporters. Custom filters did have to be designed for mCherry to shift it further away from Topaz for better optical separation (please see material and methods for filter sets used). The disadvantage of fluorescent protein reporters has to do with their sensitivity compared to enzyme based reporters. Therefore, when considering candidate genes to create fluorescent protein reporters, it is best to favor genes that have higher expression levels. In low expressing genes, signal to noise can be problematic, particularly when it is desirable to view gene expression in adult animals where tissues generally have higher levels of autofluorescence. Among the different fluorescent proteins, our experience has shown that YFP's have the best signal to noise, followed by RFP's, and lastly CFP's.

The generation of transgenic animals from this construct has been informative in regard to how one should approach future designs of multi-reporter animals. We favored the use of BAC engineered reporters because of their very large size allowing for a truer representation of endogenous gene expression. At the same time, it was hoped that the large genomic DNA regions would also contain the appropriate DNA sequences to insulate one gene from the other. Our preliminary analysis of the multiple reporter genes substantiates that their expression appears to accurately reflect their endogenous genes expression, respectively. While we do not have any definitive evidence, our data also suggests the concept of retaining sequences that would appropriately insulate one gene from another may have been erroneous. The transgenic lines from this study that retained an intact transgenic construct had low to undetectable levels of *Trap *reporter expression, while line 28-2-2 had the highest level of *Trap *expression, the lowest transgene copy number, and a fragmented transgenic construct. Given this evidence, we believe it is likely a positional effect was created by linking the *Trap *gene next to the *Dmp1*-*Ibsp *genomic fragment. However, we believe the imposed dampening effect on *Trap *expression is likely a result of its drastically different regulation relative to *Dmp1 *and *Ibsp*, being expressed in an entirely different cell type, the osteoclast. Had we chosen a different gene within the osteogenic lineage, we may have had fewer problems. A second variable in addition to gene choice is the size of genomic fragment. The region of *Trap *that we linked onto the *Dmp1*-*Ibsp *fragment was ~37 KB, which with respect to gene regulation is still relatively small and likely to be subject to positional effects. This mini-gene genomic fragment was chosen because we wanted to exclude the genes adjacent to *Trap *in the DNA construct and it was an ideal size for linkage. Disregarding the dampening effect, *Trap *reporter expression in line 28-2-2 appears to be accurate, as it is identical to the tartrate resistant acid phosphatase staining. At the same time, we cannot exclude the possibility that some of the regulatory elements for *Trap *are missing from the 37 KB genomic DNA fragment and their absence could also be a possible explanation for our results.

## Conclusion

While we speculate that gene choice and genomic DNA fragment size are critical variables that can contribute to positional effects, it is desirable not to be limited by these constraints. A potential solution to the positional effect problem is the use of insulator elements that retain both enhancer blocking and barrier properties. It would be ideal to use the native insulator that normally controls the endogenous gene. With recent whole genome mapping of gene boundaries, in the near future it may be possible to incorporate these regions into the transgenic construct design [21,22]. However, while the pace of understanding global regulator mechanisms that regulate genomic DNA has dramatically increased, in depth knowledge of how insulators work is limited to a relatively small group with most elements remaining poorly defined. With that in mind, an existing solution is the application of well characterized insulators such as the β-Globin HS4 insulator, which has been successfully used to insulate standard transgenic constructs [23,24]. It may be feasible to engineer BAC Linking vectors with insulator elements that could also potentially serve a secondary function as a common homology arm to link different genomic fragments together. Future studies will incorporate the use of well defined insulator elements in BAC linking vectors to assemble multi-reporter gene DNA constructs.

## Methods

### DNA Constructs and Reagents

BAC Clones RP23-412o5 and RP23-395m11 were obtained from Children's Hospital Research Institute (CHORI). BACLink -GM and -SP linking vectors were generously provided by Claire Huxley [10]. pLD53-SCAEB recombination vector was generously provided by Shiaoching Gong [8] and the mini lamda vector was generously provided by Donald M. Court [19]. Homology Arms were amplified using Pfx DNA polymerase (Invitrogen) and amplified using an icycler (Biorad).

### Cloning Homology Arms into BACLinking Vectors

*Trap *homology arm 3 was amplified using oligos SPA3 5'*TRAP *(ACACCTCGAGTTGTCACTTACCAGGCAAGCTGTGACA) containing an Xho1 site and SPA3 3'*TRAP *(TCTCGGATCCTTCTGTCGACGGCAGAGGCAGGCGGATTTTGAGTT) containing a BamH1 and internal Sal1 site. Homology arm 3 was digested with Xho1/BamH1 and cloned into the Sal1/Bamh1 site of BacLink-SP using conventional cloning practices. *Dmp1 *homology arm 2 was amplified using the oligos SPA2 5'DMP (TCTCGTCGACCATCTATTTATACAATTGCTCACTGAG) containing a Sal1 site and SPA2 3'DMP (TCTCGGATCCCTCTATTATAAACTGCTGTGTGTCTAAG) containing a BamH1 site and subsequently cloned into the Sal1/BamH1 site of BacLink SP-*TRAP*-A3 to create BACLink SP-*TRAP*/DMP A3/A2. *Ibsp *homology arm 1 was amplified using the oligos SPA1 5'*IBSP *(TCTCCTCGAGGTCCTTTCTACAATACTTAGAAACTTAAGT) containing an Xho1 site and SPA1 3'*IBSP *(TCTCACGCGTTCTATTGGTAACCTGCCATTTTCCCTTAGA) containing a Mlu1 site. Arm1 was then cloned into the Mlu1/Xho1 site of BACLink SP-*TRAP*/DMP A3/A2 to create BACLink SP-*TRAP*/DMP/*IBSP*. This vector was then used to capture a genomic DNA fragment containing the genes *DMP1 *and *IBSP *described below. *TRAP *homology arm 4 was amplified using the oligos GMA4 5'*TRAP *(TCTCGTCGACTGGCACGTGGGATAAGTCTATGCATGT) containing a Sal1 site and GMA4 3'*TRAP *(CTCTGGATCCCAGTAGCTACCACTTGCTGGTTTTGAG) containing a BamH1 site and cloned into the Sal1/BamH1 site of BACLink-GM to create BACLink-GM *TRAP*-A4. *TRAP *homology arm 3 was amplified using oligos GMA3 5'*TRAP *(ACACCTCGAGTTGTCACTTACCAGGCAAGCTGTGACA) containing an Xho1 site and GMA3 3'*TRAP *(TCTCACGCGTGGCAGAGGCAGGCGGATTTTGAGTTCA) containing an Mlu1 site and cloned into the Xho1/Mlu1 site of BACLink-GM *TRAP*-A4 to create BACLink-GM *TRAP*-A3/A4. This vector was subsequently used to capture a genomic DNA region of the *TRAP *gene.

### Subcloning Genes into BAC Linking vectors

RP23-412o5 and RP23-395m11 containing DH10B were made electrocompetent and transformed with mini lamda [19]. Colonies were selected for on Chloramphenicol (12.5 ug/ml) and Tetracycline (10 ug/ml) LB Agar plates and grown at 32c. Resistant colonies were expanded and made electrocompetent. To prepare electrocompetent cells containing mini lamda, cells were maintained at 32c, but at the end of the growth phase were heat shocked at 42c for 15 minutes to activate the Red Recombinase System. BACLink SP *TRAP*/DMP/*IBSP *was double digested with Not1/SacI and transformed into RP23-395m11/mini lamda bacteria and selected for on spectinomycin (50 ug/ml) resistant LB Agar plates. BACLink GM *TRAP *A3/A4 was digested with Xho I and transformed into RP23-412o5/mini lamda bacteria and selected for on gentamicin (5 ug/ml) resistant plates. Recombinants were initially screened by colony PCR where oligos were designed flanking homology arms, one being present in the vector and the other being present on the other side of the homology arm. For the *DMP1*/*IBSP *genomic DNA fragment oligos SP2 (GCCCTACACAAATTGGGAGA) and DMPR (ACTCTTTCCTTAAAGATATCAATTTAC) were used to screen the *DMP1 *end and Belo2658 (TTTGTCACAGGGTTAAGGGC) and *IBSP*R (TCTGCTGATGTGTCCACCAGCACTAAG) were used to screen the *IBSP *end. For the *TRAP *genomic DNA fragment oligos Belo 2658 and *TRAP*-A3R (AATTACAGATTTGTGAGATAGTCACAC) were used to screen the arm 3 end and GM5'end (CGTAACATCGTTGCTGCTGCGTAACAT) and *TRAP*-A4R (CCGCAGATGGACTTCTGTCCAGCTGAG) were used to screen the arm 4 end. Potential BAC subclones positively identified by PCR were further verified by restriction digested followed by FIGE.

### Cloning of Homology Arms into pLD53 Vectors

Homology Arms were cloned into pLD53 Vectors using standard cloning practices. Homology arms were PCR amplified using PFX polymerase from RP23-412o5 and RP23-395m11 BAC clones. Two homology arms were amplified for each gene (referred to as arms A, B, C, D, E, & F). Primer sequences are as follows: *TRAP*Box E5'Mlu (TCTCACGCGTGAAGTCCAGTGCTCACATGAC), *TRAP*BoxE3' Not (AGTGGCGGCCGCCCATGAATCCATCTGTGAGGAAGAGAG), *TRAP*BoxF5'PAC1 (GTGCTTAATTAAGCGCTGACTTCATCATGTCTC), *TRAP*BoxF3'PAC1 (GTGCTTAATTAAGACATACACACAGACACACAC), *IBSP*BoxA5'Mlu (TCTCACGCGTCTGTGAAGTATTCAAGGTACTC), *IBSP*BoxA3'NHE (CTAGCTAGCTGCAATTTCTTCTG CAATTGAAG), *IBSP*BoxB5'BSIW1(GATCGTACGGACTGCTTTAATTTTGCTCAGC), *IBSP*BoxB3'PAC1 (GTGCTTAATTAATATCACTGGCTCTACTGTCAGTC), *DMP1*BoxCMlu (TCTCACGCGTGCTTCTGAG TTGGTGGAGAGATAC), *DMP1*BoxC3'NHE1(CTAGCTAGCGGATGCGATTCCTCTACCTGTAATGAAAG), *DMP1 *BoxD5'BSIW1/Cla1 (CATCGTACGATCGATGACTGTCATTCTCCTTGTGTTCC) *DMP1*BoxD 3'BSIW1(GATCGTACGGGATCGTAGTTCATACTACTTAC) Amplified products were run through a PCR clean up column (Qiagen), restriction digested and gel purified (Zymogen). Plasmids were digested and then briefly treated with Calf Intestinal Alkaline Phosphatase (New England Biolabs) followed by phenol chloroform extraction and precipitation. Vector and inserted were mixed, ligated at room temperature for 1 hour and electroporated into PIR2 cells. Transformants were selected on LB plates with ampicillin (50 ug/ml).

### Inserting Fluorescent Proteins into Genes

After cloning in homology arms A and B, 1 ug of pLD53 was electroporated into 40 ul of electrocompetent bacteria containing the BAC clone of interest. Bacteria were grown for 1 hour in SOC media by shaking at 37c without antibiotic selection. The entire transformation suspension was plated onto LB plates with the appropriate antibiotics (ampicillin 50 ug/ml, gentamicin 5 ug/ml, spectinomycin 50 ug/ml). Ten colonies were picked and re-streaked onto a new plate and grown overnight. The next morning the ten colonies were grown in 1 ml LB media containing the appropriate antibiotic. When bacteria growth appeared, 50–100 ul of LB was taken to screen for an arm A recombination event by colony PCR. PCR primers were designed to flank the A homology arm. Gene specific sense primers were: *TRAP*recomA (ACACATTACCATCAGACCCTG), *IBSP*recomA (GTCTGATACCTCCGAAGAGCTCAC), *DMP1*recomA (GTTAGGTTGCTGTGTAATACTGGC). Fluorescent protein antisense primers were: CT genotype (GTTTACGTCGCCGTCCAGCTCGACCAGGAT) for ECFP and Topaz fluorescent proteins and mCherry genotype (GCACCTTGAAGCGCATGAACTCCTTGATGA) for mCherry fluorescent protein. The rest of the bacteria culture was grown to confluence and minipreps were carried out. BAC clones were digested with Sal I to verify integration of pLD53 vector (pLD53 introduces a SalI site). Two positive integrants as determined by colony pcr and FIGE were further processed for backbone resolution. Two colonies from each clone were picked from the original re-streaked plate and grown for one hour with shaking in LB media without ampicillin, but in the presence of gentamicin or spectinomycin. 10–100 ul of media was spread onto TG plates and grown overnight at 37c. 40 colonies were picked and replated onto LB plates containing the appropriate antibiotic (no amplicillin). To enrich for resolved clones gentamicin/ampicillin or spectinomycin/ampicillin plates were also used as a replica of the 40 clones. Small colonies were favored over larger colonies when picking clones. Twenty ampicillin sensitive colonies were grown initially screened by colony PCR to verify the presence of the fluorescent protein reporter. Diagnostic restriction digests and FIGE was further carried to verify the genomic integrity of the clones and confirm pLD53 backbone resolution. Moreover, primers flanking the B homology arm were also used in combination with recomBoxA oligos to confirm resolution: *TRAP*recomB (CATAATCTGTCCTCTGGCCTG), *IBSP*recomB (GAACAGCTGGCTGACAGCACTGAATCAAC), *DMP1*recomB (TCCAGTTACACCACATAG GAATTG).

### Linking Genes of Interest

The bacteriophage Red recombinase system was introduced into BACLink-SP containing DMP1-mCherry and IBSP-Topaz reporter genes by transformation of mini lamda and bacteria cells were made electrocompetent as described above. 10 ug of BACLink-GM TRAP-ECFP was digested with I-PPO1, phenol/cholorform extracted, precipitated, and resuspended in TE. 2 ug of digested BAC was transformed into competent bacteria containing SP-DMP1mCherry/IBSP-Topaz. Transformed colonies were selected for on LB gentamicin plates (5 ug/ml). Clones were initially screen by PCR using primers BL-TRAP (ACAGATTTGTGAGATAGTCACACAATTC) and BL-DMP1 (GACAATGTTTGCAGACTATGAATGAAG) that flank the linked genomic region. PCR positive clones were further verified by diagnostic restriction digest.

### Generation of Transgenic Animals

The linked BAC construct was purified from 250 mls of bacteria culture using a large construct kit (Qiagen). 10 ug of BAC was linearized with I-PPO1 restriction enzyme for 2 hours and was further purified on a CL-4B sepharose (Sigma) column that was pre-equilibrated with injection buffer (10 mM Tris pH7.5, 0.1 mM EDTA, 100 mM NaCl). Twelve 200 ul fractions were collected and BAC DNA was quantified using a pico green DNA assay (Molecular Probes) and/or using a Nanodrop spectrophotometer (Thermoscientific). Pronuclear injection was carried out at the UCONN Health Center Gene Targeting and Transgenic Facility (GTTF).

### Preliminary Transgenic Characterization

For imaging of transgene expression in spine, 6 week old females were sacrificed in accordance with our animal care protocols and tissues were dissected and fixed in 4% paraformaldehyde for 4 days, decalcified in 28% sodium free EDTA for 5 days, immersed in 30% sucrose/1 mM MgCl_2 _for 1 day. Decalcified tissue was frozen embedded and cryosectioned on to cryofilm type IIC tape (Finetec). Sections were imaged on a Zeiss Z.1 Observer inverted microscope. Filter sets used for imaging were purchased from Chroma Technology and are as follows: (HQ500/20 Ex, HQ 535/30 Em, Q515lp beam splitter) for Topaz (YFP), (D436/20 Ex, D480/40 Em, Q455dclp beam splitter) for ECFP, (HQ577/20 Ex, HQ640/40 Em, Q595lp beam splitter) for mCherry. *TRAP *staining was carried out after imaging bone sections with for ECFP using the Acid Phosphatase Kit (Sigma).

### Estimating Transgene Copy Number

Tails were clipped from three animals of each mouse line and wild type animals. Genomic DNA was prepared using a DNAeasy Blood and Tissue Kit (Qiagen) and real time PCR was carried out on an I-Cycler (Biorad) for the *Trap *gene 5'TRAP (GTCTGTGGAACTGACGGCTGTAGATGGCTA) 3'TRAP (AGTGGCGGCCGCCCATGAATCCATCTGTGAGGAAGAGAG) and normalized to the Biglycan gene 5'-Biglycan (CAGAGCTTACACCCACTAACATACTC) 3'-Biglycan (CTCCGAAGCCCATAGGACAGAAGTCA).

Fold difference of transgenic mouse lines was compared to wild type CD1 mice and transgene copy number was estimated based on this fold difference.

## Abbreviations

BAC: Bacterial Artificial Chromosome; Rec A: Recombinase A; FP: Fluorescent Protein; *TRAP*: Tartrate Resistant Acid Phosphatase; *DMP1*: Dentin Matrix Protein 1; *IBSP*: Integrin Binding Sialoprotein; ECFP: Enhanced Cyan Fluorescent Protein; KB: Kilobase; SP: Spectinomycin; GM: Gentamicin; AMP: Ampicillin; CHORI: Children's Hospital Oakland Research Institute; BP: Base Pair.

## Authors' contributions

PM conceptualized the study, supervised the study, assembled the transgenic construct along with MLS, and wrote the manuscript. YL maintained the mice and carried out tissue histology, genotyping and real time PCR. SG provided the pLD53-SCAEB RecA/SacB recombination vector. ACL and DWR provided support and consulting on this project. All authors read and approved this manuscript.
